# The effectiveness of peer and community health worker-led self-management support programs for improving diabetes health-related outcomes in adults in low- and-middle-income countries: a systematic review

**DOI:** 10.1186/s13643-020-01377-8

**Published:** 2020-06-06

**Authors:** Mahmoud Werfalli, Peter J. Raubenheimer, Mark Engel, Alfred Musekiwa, Kirsten Bobrow, Nasheeta Peer, Cecilia Hoegfeldt, Sebastiana Kalula, Andre Pascal Kengne, Naomi S. Levitt

**Affiliations:** 1 Chronic Disease Initiative for Africa, Cape Town, Western Cape South Africa; 2 Department of Medicine, Faculty of Health Science, University of Cape Town, Observatory, Cape Town, Western Cape 7935 South Africa; 3 Chronic Diseases of Lifestyle Research Unit, Durban, Durban, South Africa; 4 Department of Psychiatry, University of Oxford, Oxford, UK; 5 Department of Geriatric Medicine, Faculty of Health Science, University of Cape Town, Cape Town, Western Cape South Africa; 6 South African Medical Research Council, Cape Town, South Africa; 7 Division of Endocrinology and Diabetes, Chronic Diseases Initiative for Africa (CDIA), Department of Medicine, University of Cape Town, J 47 Room 86, Old Groote Schuur Hospital Building, Cape Town, South Africa

## Abstract

**Objective:**

Community-based peer and community health worker-led diabetes self-management programs (COMP-DSMP) can benefit diabetes care, but the supporting evidence has been inadequately assessed. This systematic review explores the nature of COMP-DSMP in low- and middle-income countries’ (LMIC) primary care settings and evaluates implementation strategies and diabetes-related health outcomes.

**Methods:**

We searched the Cochrane Library, PubMed-MEDLINE, SCOPUS, CINAHL PsycINFO Database, International Clinical Trials Registry Platform, Clinicaltrials.gov, Pan African Clinical Trials Registry (PACTR), and HINARI (Health InterNetwork Access to Research Initiative) for studies that evaluated a COMP-DSMP in adults with either type 1 or type 2 diabetes in World Bank-defined LMIC from January 2000 to December 2019. Randomised and non-randomised controlled trials with at least 3 months follow-up and reporting on a behavioural, a primary psychological, and/or a clinical outcome were included. Implementation strategies were analysed using the standardised implementation framework by Proctor et al. Heterogeneity in study designs, outcomes, the scale of measurements, and measurement times precluded meta-analysis; thus, a narrative description of studies is provided.

**Results:**

Of the 702 records identified, eleven studies with 6090 participants were included. COMP-DSMPs were inconsistently associated with improvements in clinical, behavioural, and psychological outcomes. Many of the included studies were evaluated as being of low quality, most had a substantial risk of bias, and there was a significant heterogeneity of the intervention characteristics (for example, peer definition, selection, recruitment, training and type, dose, and duration of delivered intervention), such that generalisation was not possible.

**Conclusions:**

The level of evidence of this systematic review was considered low according to the GRADE criteria. The existing evidence however does show some improvements in outcomes. We recommend ongoing, but well-designed studies using a framework such as the MRC framework for the development and evaluation of complex interventions to inform the evidence base on the contribution of COMP-DSMP in LMIC.

## Introduction

Over the past decade, diabetes prevalence has risen faster in low- and middle-income countries (LMIC) than in high-income countries (HIC) [1]. Currently, about 80% of people with diabetes worldwide live in LMIC, and projections suggest that some of these countries will experience more than a twofold increase in the number of people affected over the next 20 years [1]. People living with diabetes need not only medical treatment from their health care providers; equally important is self-management and sustaining complex self-care behaviours. These behaviours (under the umbrella of “self-management”) include following complicated medication regimens and often embarking on significant lifestyle changes in diet and exercise programs, monitoring and responding to symptoms, and coping effectively with stress [2–4].

Evaluation of diabetes self-management programs has shown improved health outcomes and reduced utilisation of health services [5–7]. However, without continuous support, many adults will not succeed in managing their condition well, leading to worse health outcomes, including expensive hospitalisations and avoidable complications [8]. It is critical for health care providers and the settings where they work to have the resources and a systematic referral process to ensure that patients with diabetes consistently receive both diabetes self-management education (DSME) and diabetes self-management support (DSMS). DSME is defined as the ongoing procedure of facilitating the knowledge, skill, and ability necessary for diabetes self-care, while DSMS is defined as activities assisting the diabetic patient in implementing and sustaining the behaviours needed to manage his/her condition on an ongoing basis beyond or outside of formal self-management training. The type of support provided can be behavioural, educational, psychosocial, or clinical [9]. The initial DSME is typically provided by a healthcare professional, whereas ongoing support can be provided by personnel without a formal health tertiary education [9]. However, health resources, infrastructure, and well-equipped health staff are often limited in LMIC which complicate the delivery and sustainability of DSME and DSMS [10].

A potential solution for delivering diabetes self-management support could be task-shifting. This is the process whereby tasks are moved, where appropriate, to less specialised health workers and is thus expected to reduce health care expenses while improving health care coverage [11]. Lay health workers from the community such as ‘patient-peers’ or community health workers (CHW) are ideally suited for such task-shifting since existing research suggests that such programs are an effective and relatively inexpensive means to help patients manage chronic conditions [12, 13]. Furthermore, these programs have been recommended by the World Health Organization review committee on peer support in diabetes as a resourceful way for diabetes management [14].

Existing COMP-DSMPs involve two types of closely related lay health care workers: ‘patient-peers’ (here called ‘peers’) and community health workers (CHW). For the purpose of this review, Dennis’s [15] comprehensive definition of peer support, as used in a recent Cochrane review [16], is employed. Dennis defines peer support as ‘provision of emotional, appraisal and informational assistance by a created social network member who possesses experiential knowledge of a specific behaviour or stressor and similar characteristics as the target population, to address a health-related issue of a potentially or stressed focal person’ [15]. To possess this experiential knowledge, peers must be affected by the same condition as the patient population they serve. In the context of diabetes, peers often have diabetes themselves or have a family member with diabetes. Their support can help metabolic control by sharing, discussing, identifying, and facilitating behaviours, which can improve diabetes self-management and overcome obstacles to care and self-care [17].

CHWs constitute another form of lay health workers based in the local community. WHO defines CHWs as health workers without a tertiary education health certificate, who are members of the communities where they work, and are supported by the health system, although not necessarily part of its organisation [18]. In contrast to peers, CHWs do not necessarily have the experiential knowledge of being a patient. Yet, similar to peers, CHWs speak the language and share culture and community with the patients with whom they work. Like peer support, CHW support varies widely across different contexts and may include both self-management support and direct patient care [19, 20]. Furthermore, both CHW and peer-support interventions (here collectively referred to as COMP-DSMPs) differ in the extent and type of formal training that peers/CHWs receive, in whether peers/CHW are paid members of a healthcare team or volunteers, in the type and extent of time commitment required of the peers/CHW, and in the principal method of peer support (for example, face-to-face contact versus telephone contact) [21].

A considerable body of evidence from well-designed RCTs, mainly in HIC, demonstrates improved clinical and behavioural outcomes such as glycaemic control in diabetes populations receiving peer/CHWs support [22–31]. This is further supported by several systematic reviews. A systematic review conducted by Zhang et al. suggested that home-visit-intervention and curriculum-combined-reinforcement-intervention performed by peers had a better effect on improving glycaemic control compared to conventional care [32]. Furthermore, a systematic review by Norris et al. reported positive changes in lifestyle and self-care in some studies of CHW-led interventions for diabetes self-management. Although limited data on economic outcomes is available, several studies demonstrated a reduction in health care expenses as a result of the CHW-led intervention [33].

At present, there are no systematic reviews of peer/CHW support programs for diabetes focusing on LMICs. Furthermore, most systematic reviews to date have not applied a standardised framework for analysing and evaluating the implementation strategies across studies. Systematic categorisation and assessment of implementation outcomes are critical for assessing whether an implementation strategy has been applied successfully since an intervention will not be successful unless both the implementation of the strategy in a given context and the components of the strategy itself are effective [34]. In the case of failure, it is essential to know if this was due to the intervention being ineffective in the new setting (intervention failure), or if an intervention was deployed incorrectly (implementation failure) [35]. To bridge this gap in the existing literature, this systematic review aims to employ a standardised taxonomy for analysing and evaluating COMP-DSMPs implementation strategies in LMIC for diabetes self-management. We strive to answer the following questions: What are the effects of COMP-DSMPs on the clinical and behavioural outcomes of adults with diabetes, and how consistent are those effects across existing studies? What were the program designs used and how were the implementation outcomes assessed?

## Methods

A full study protocol was developed and published in a peer-reviewed journal [36]. This systematic review has been modified from the protocol. Firstly, a meta-analysis was precluded by the quality of the included studies. Secondly, the research question addressing ‘how COM-DSMP can help improving quality of diabetes care’ has been modified to addressing ‘the program designs used, and the implementation outcomes assessed’ since there was insufficient information on the quality of care in the included studies. Thirdly, we have added the Risk of Bias in Nonrandomized Studies of Interventions (ROBINS-I) tool for non-randomised studies [37]. Finally, implementation taxonomy frameworks by Proctor et al. have been adopted for the analysis and evaluation of the implementation strategies. This review follows the Preferred Reporting Items for Systematic Reviews and Meta-Analyses (PRISMA) [38] and is registered with the International Prospective Register of Systematic Reviews [registration number CRD42014007531].

### Search strategy

We searched the Cochrane Library, MEDLINE via PubMed, SCOPUS, CINAHL, PsycINFO, and Web of Science databases for studies published between 1 January 2000 and 31 December 2019, which evaluated COMP-DSMPs in adults with diabetes in LMIC. Drawing on a combination of free-text search terms, Medical Subject Headings, and database-specific subject headings, we developed a sensitive search strategy for multiple electronic databases (Additional file 1), combining synonyms for ‘diabetes’, ‘peer support’, ‘community health worker’, ‘intervention’, and ‘LMIC’.

Other database resources such as Google Scholar, WHO, Peer for Progress, International Clinical Trials Registry Platform, Clinicaltrials.gov, Pan African Clinical Trials Registry (PACTR), and HINARI (Health InterNetwork Access to Research Initiative) for LMIC were searched. We similarly explored the reference lists of key articles and journals.

### Selection of studies

Titles and/or abstracts of studies were identified using the search strategy, and those from additional sources were screened independently by two reviewers (MW, PR). They individually assessed the eligibility of the articles first based on the title and abstract and later on full text. Any disagreement between the two reviewers was resolved through discussion with a third author (NSL) on the study team.

### Inclusion criteria

- Types of studies: Studies that measured the effects of COMP-DSMPs in randomised controlled trials (RCTs), non-randomised controlled trials, and quasi-randomised controlled trials were included. The quasi-randomised controlled trials included controlled studies with a comparison group and uncontrolled studies with ‘before and after’ study designs. We included both controlled and non-controlled before and after studies because they are accepted research designs for improvement strategies and are widely used, especially so in LMIC where the resources are not available to conduct RCTs [35].
- Types of participants/population: Only studies from LMIC based on the World Bank classification of country income groups were included [39]. Study participants had to be ≥ 18 years of age and have either type 1 or type 2 diabetes, but not gestational diabetes nor diabetes due to other causes. We included type 1 and type 2 diabetes because several studies from LMIC do not differentiate between these two diagnoses.
- Types of interventions: Studies that reported contact with an individual or a group of peers (paid or voluntary) offering COMP-DSMP with a minimum follow-up period of 3 months were included. Peers could be CHW, peer leader, lay health advisor, lay health educators, or peer coaches. Peer support that was exclusively telephone- and web-based was excluded. Interventions led or facilitated by a professional (or non-peer) were included, providing that the focus of the intervention was to provide peer-to-peer interaction. Studies in which peer support was part of a multicomponent/complex intervention, where the effects of the peer support element could not be isolated, were excluded.
- Types of control/comparator groups: Studies in which the control/comparator group received usual care or professional health worker-led diabetes self-management support (and not peer support) with a follow-up period of 3 months or more were included.
- Types of outcomes: Studies that reported at least one of the following outcomes were included. *Behavioural—*such as physical activity/fitness, glucose monitoring, adherence to medication, improved nutrition, and self-care. *Psychological—*such as self-efficacy, knowledge, attitudes, quality of life, confidence, self-esteem, well-being, vitality, social functioning, and coping, as assessed by validated measures. *Clinical—*such as fasting and random blood sugar levels, glycated haemoglobin (HbA1c), cholesterol, blood pressure, body mass index (BMI), symptoms of hypoglycaemia and hyperglycaemia, and hospitalisations or clinical visits.
- Language: Restricted to English
- Time restriction: We decided to restrict the search to the period following the changes to the diabetes diagnostic criteria in 1999 based on the WHO Expert Committee on Diagnosis and Classification of Diabetes. Thus, all studies from 1 January 2000 to 31 December 2019 were eligible if the other inclusion criteria were met.

### Assessment of risk of bias of studies

Four reviewers (MW, PR, NP, and KB) independently evaluated and reported on the risk of bias as described in the Cochrane Handbook for Systematic Reviews of Interventions according to the criteria and associated categorisations contained therein for randomised trials and using the Risk of Bias in Nonrandomized Studies of Interventions (ROBINS-I) tool for non-randomised studies [37, 40]. A consensus was reached after discussion and consultation with another reviewer (ME).

The quality of evidence for the outcomes was assessed by the Grading of Recommendations Assessment, Development and Evaluation (GRADE) criteria of risk of bias, inconsistency, indirectness, imprecision, and study limitation. The quality assessment was categorized as high, moderate, low, or very low.

### Data extraction and synthesis

The following data were extracted independently by three reviewers (MW, PR, and NP): author, year of publication, geographic region, study design, description of the intervention (including process, cost of programme, cost-effectiveness if available, context of intervention (i.e. primary health facility), details about group leader (demographics, training, professional status, etc.), details about participants (including number of each group, baseline health information, demographic characteristics), length of intervention and follow-up, definition of peer used, and health outcomes. The data abstraction forms based on the Cochrane Consumers and Communication Review Group’s Data Extraction Template for Cochrane Reviews were modified to fit this review (Additional file 2). A consensus was reached by discussion and consultation with other reviewers (ME, NSL) where necessary.

### Taxonomy for analysing implementation strategies

The taxonomy used to investigate and evaluate the implementation strategy of each study is based on previously published conceptual frameworks by Proctor et al. [41, 42]. Proctor et al. [41] propose guidelines for naming, defining, and operationalising implementation strategies in terms of seven dimensions: actor, the action, action targets, temporality, dose, implementation outcomes addressed, and theoretical justification. For this review, we categorised and analysed the included studies by applying six of the proposed dimensions (Table 2), while ‘implementation outcomes addressed’ are separately analysed by the taxonomy proposed by Proctor et al. [42] (Table 3). Although the conceptual framework is intended for researchers planning implementation strategies, it allows for systematic investigation, evaluation, and comparison of the nature of the implementation strategies in the included studies. Furthermore, it enables investigation of whether the studies suffer from commonly reported problems in the current implementation research such as inconsistent labelling, poor descriptions, and unclear justification for specific implementation strategies. The *actors* are defined as the stakeholder delivering the strategy; the *actions* are defined as those actions enacted by the actors; *action targets* are the population *targeted* by the intervention and how the actions are supposed to impact this population; *temporality* is defined as the phased nature of implementation meaning at which stage was the strategy used relative to other stages; *dose* is defined as the frequency and intensity of the implementation strategy such as the amount of time spent with an external facilitator; and *theoretical justification* is defined as the justification or rationale for the implementation strategy, which can be theoretical, empirical, and/or pragmatical.

### Taxonomy for evaluating the implementation strategies

We systematically investigated whether studies reported on the eight implementation outcomes prescribed by Proctor et al. [42]: *acceptability* (i.e. the perception among stakeholders that an intervention is agreeable), *appropriateness* (i.e. the perceived fit or relevance of the intervention in a setting or for a particular target audience or issue), *feasibility* (i.e. the extent to which an intervention can be carried out in a specific setting or organisation), *adoption* (i.e. the intention, initial decision, or action to try to employ a new intervention), *penetration* (i.e. the degree to which the population who is eligible to benefit from an intervention actually receives it), *sustainability* (i.e. the extent to which an intervention is maintained or institutionalised in a given setting), *implementation costs* (i.e. the incremental cost of the delivery strategy), and *implementation fidelity* (i.e. the extent to which an intervention is delivered as planned). We assessed implementation fidelity (IF), using the models of Carroll et al. [54].

### Statistical analysis

Comparisons between groups for continuous outcomes were conducted using mean differences (MD) and their corresponding 95% confidence intervals and *p* values. For binary outcomes, proportions or percentages were compared using chi-square tests. Most studies used regression analyses to compare outcomes between the study groups with adjustments for multiple comparisons, baseline, and confounding variables, and in these cases, we used the *p* values reported by the study authors. However, differences in study designs, outcomes, the scale of measurements, and measurement times precluded meta-analysis. We, therefore, provide a narrative summary of the findings across studies.

## Results

### Summary of the searches

**Fig. 1:**
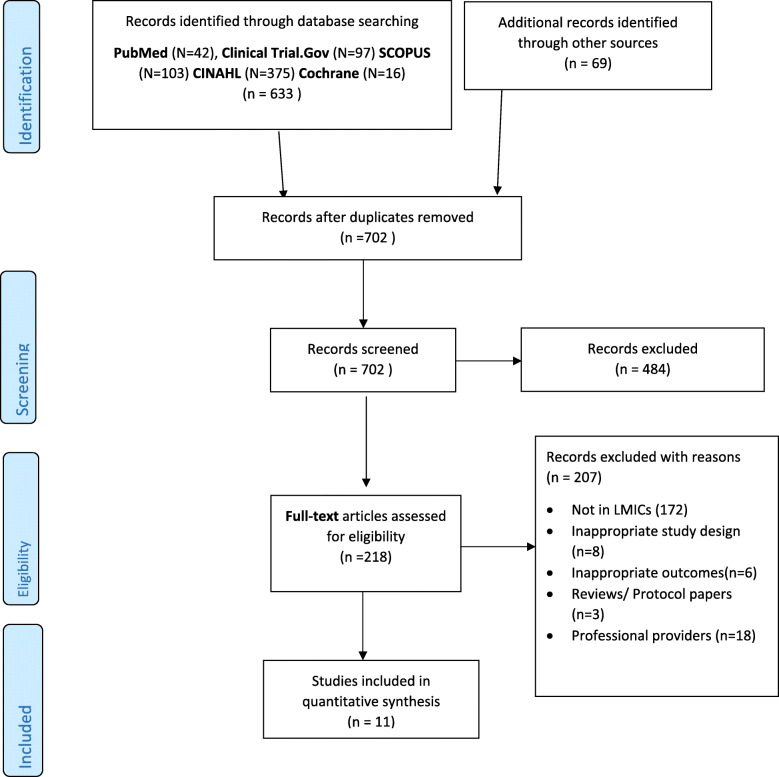
The PRISMA flow diagram is depicting the flow of information through the different phases of the systematic review

A flow consort diagram of the studies selected for inclusion is summarised in Fig. 1. A total of 702 records were identified from searches. After removal of duplicates and title and abstract screening, 218 articles were selected for further evaluation via full text; of these, 207 full-text articles were excluded, 172 were not from LMIC, 18 were led by professional providers, 8 had inappropriate study designs, and 3 were reviews or protocol papers. Thus, only eleven studies met the inclusion criteria and were included in the systematic review. These studies were published in the period from 2008 to 2019 based on patient populations in the following countries: South Africa (*n* = 2), Cameroon (*n* = 1), Uganda (*n* = 1), China (*n* = 2), Cambodia (*n* = 1), Argentina (*n* = 1), Guatemala (*n* = 1), Jamaica (*n* = 1), and Mali (*n* = 1). The included studies comprised four RCTs [50–53], one non-randomised parallel arms intervention study [43], and six pretest-posttest studies [44–49]. The sample sizes in the studies varied from 19 to 2714; in total, there were 6090 participants in this review. Seven studies investigated support provided by peers, while the remaining three studies explored support provided by CHWs.

### Quality of included studies

A summary of the risk of bias of included RCT selected studies by using the Cochrane tool is shown in Table 1. Two studies (Zhong et al. [51] and Gagliardino et al. [52]) failed to report details about random sequence generation and also details of allocation concealment. Only one study (Debussche et al. [50]) described the use of blinding of participants and investigators, three studies (Zhong et al. [51] and Gagliardino et al. [52] and Mash et al. [53]) had high risk of bias in blinding of participants and personnel, and other studies did not report the detailed information. The outcomes assessor was reported to be blinded in one study (Debussche et.al 2018). Only one study (Mash et al.,2014) described the reasons for participants’ withdrawals.

**Table 1:** Risk of bias for included RCTs

Authors, year	Selection bias, random sequence generation	Selection bias (allocation concealment)	Detection bias, blinding (outcome assessment)	Attrition bias, incomplete outcome data	Reporting bias, selective reporting	Performance bias, blinding (participants and personnel)
Debussche et al. 2018 [50]	Low	Low	Low	Low	Low	Low
Zhong et al. 2015 [51]	NCR	NCR	High	High	Low	NCR
Gagliardino et al. 2014 [52]	NCR	NCR	High	NCR	Low	High
Mash et al. 2014 [53]	Low	NCR	High	Low	Low	High

*NCR* no clear risk

A summary of the risk of bias for the non-RCTs by using ROBINS-I tool is illustrated in Table 2 and was applied retrospectively. A serious risk of bias was assessed for all seven studies for the following domains: confounding, selection of participants, classification of interventions, and measurement of outcomes, and for four studies for selection of the reported results. As a result, our overall judgement was that all studies had serious risks of bias.

**Table 2:** Results of the assessment of risk of bias in included non-randomized studies by using the ROBINS-I assessment tool

Study ID	1. Bias caused by confounding	2. Bias caused by selection of participants	3. Bias caused by classification of interventions	4. Bias caused by deviations from intended interventions	5. Attrition bias caused by missing data	6. Detection bias caused by measurement of outcomes	7. Reporting bias caused by selection of the reported results	Overall judgement
Assah et al. [43]	Serious	Serious	Serious	Low	No information	Serious	Serious	Serious
Baumann et al. [44]	Serious	Serious	Serious	Low	Low (information on reasons for missing data provided)	Serious	Serious	Serious
Eggermont [45]	Serious	Serious	Serious	Low	Low (information on reasons for missing data provided)	Serious	Moderate	Serious
Rotheram-Borus et al. [46]	Serious	Serious	Serious	Low	Serious	Serious	Serious	Serious
Shen [47]	Serious	Serious	Serious	Low	No information	Serious	Low	Serious
Less et al. [48]	Serious	Serious	Serious	Low	Low (information on reasons for missing data provided)	Serious	Low	Serious
Micikas et al. [49]	Serious	Serious	Serious	Low	No information	Serious	Serious	Serious

In the GRADE analysis, two studies were judged as having moderate quality [51, 53] (Table 3). The results did not fully answer our question, as well as showing imprecision for not properly implementing the COMP-DSMPs; therefore, considering the overall risk of bias, only one study [50] was judged as having high-quality evidence for the question of this systematic review.

**Table 3:** Assessment of quality of evidence (GRADE) in the included studies

Study ID	Study design	Study limitation	Inconsistency	Indirectness	Imprecision	Bias	Overall quality
Debussche et al. [50]	**RCT**	**√**	**√**	**√**	**√**	**√**	**++++**
Zhong et al. [51]	**RCT**	**√**	**X**	**√**	**√**	**X**	**+++**
Gagliardino et al. [52]	**RCT**	**X**	**X**	**√**	**√**	**X**	**++**
Mash et al. [53]	**RCT**	**√**	**X**	**√**	**√**	**X**	**+++**
Assah et al. [43]	**Non-RCT with control**	**X**	**X**	**√**	**√**	**X**	**++**
Baumann et al. [44]	**UCBA (one-group)**	**X**	**X**	**√**	**√**	**X**	**++**
Micikas et al. [49]	**UCBA (one-group)**	**√**	**X**	**√**	**X**	**X**	**++**
Eggermont [45]	**UCBA (one-group)**	**X**	**X**	**√**	**√**	**X**	**++**
Rotheram-Borus et al. [46]	**UCBA (one-group)**	**X**	**X**	**√**	**√**	**X**	**++**
Shen [47]	**CBA (comparison group)**	**√**	**X**	**√**	**√**	**X**	**++**
Less et al. [48]	**CBA (comparison group)**	**√**	**X**	**√**	**√**	**X**	**++**

√ no serious limitations; *X* serious limitations, for overall quality of evidence: + very low; ++ low; +++ moderate; ++++ high; *RCT* randomize control trials; *CBA* controlled before and after studies; *UCBA* uncontrolled before and after

### Taxonomy of implementation of peer support strategies

Table 4 illustrates the taxonomy of the peer/CHW support implementation strategies in the included studies based on the framework by Proctor et al. [41].

**Table 4:** Taxonomy of implementation of peer support strategies in LMIC by mode of delivery

Study ID and design	Country	Actors Who delivers the strategy?	Actions Which actions do the actors enact?	Targets of action Who/what are the actors attempting to impact?	Temporality At which phase is the strategy used?	Dose At which frequency and intensity is the strategy used?	Justification Which (theoretical, empirical, pragmatic) justification/rationale is provided for the choice of implementation strategy?
Assah et al. 2015 Non-RCT [43]	Cameroon	**Peers—**volunteers with diabetes selected based on their compliance with treatment, good glycaemic and metabolic control, and their experience. Received 2-day training workshop.	**Actions—**actors led group meetings on self-management and conducted personal and telephone-based support.	**Targets—**actions were aimed at improving self-care behaviours; knowledge; clinical outcomes (glycaemic levels, blood pressure, lipids); providing emotional and social support for adults with poorly controlled T2DM.	N/A	**Dose—**Group meetings monthly for 6 months. Personal + telephone encounters—5 monthly over 6 months.	**Justification—**Research shows that peer-support care models provide low-cost, flexible means to supplement formal health care support for chronic diseases.
Baumann et al. 2015 Pre-post quasi-experimental study [44]	Uganda	**Peers** (called champions)**—**people with diabetes who were able to read and speak English and receive 2 days training in communication, emotional support, and assistance with daily management. Other selection criteria not specified.	**Actions—**actors were matched with patient peers and provided emotional support and assistance with daily management through facilitating personal and telephone.	**Targets—**actions were aimed at improving diabetes self-care behaviours, glycaemic control, social support, emotional well-being, and linkage to health-care providers for adults with T2DM.	N/A	**Dose—**Contact between peers and partners (telephone/in person) at least once a week over 4 months.	**Justification—**WHO suggests that peer support is a promising approach toward achieving self-care goals in a developing world setting with shortage of health workers, which is supported empirically.
Eggermont 2011 Pre/post [45]	Cambodia	**Peers—**recently recovered from years of serious illness from poor glycaemic control. Received 6 weeks of training.	**Actions—**actors educated and provided skills of self-management; supported adaptation of life-style including nutrition and daily exercise; and mediated contact to professional health staff when needed.	**Targets—**actions aimed to improve health outcomes (blood glucose, blood pressure, BMI), ability to control disease, and empower people with diabetes (some also had hypertension).	N/A	**Dose—**Classes—6 in the home of peer educator. Monitor glucose levels—twice monthly Time period not specified.	**Justification—**Peer support models are theoretically promising for resource constrained health systems and underpins patient-centeredness, supported by some empirical evidence.
Gagliardino et al. 2013 RCT [52]	Argentina	**Peers—**patients with diabetes recruited on the basis of their excellent diabetes control, self-motivation, communication and support skills. Recruited from an NGO devoted to education of people with diabetes. Received 3 days training in DSM and communication.	**Actions—**actors implemented a diabetes educational program, provided psychological and behavioural support through phone calls to patients and face-to-face interviews in small groups.	**Targets—**The actions aimed at improving and sustaining self-care behaviours and hereby clinical outcomes in adults (25–75 years) with T2DM, who had been followed for at least 2 years by physicians without major co-morbidities.	N/A	**Dose—**Educational course, 4-week program (4 modules, 90–120 min). 1 reinforcement session 6 months after. Calls—weekly for 6 months post-course, biweekly next 3 months, and monthly for last 3 months. Interviews—bimonthly for 1 year post-course.	**Justification—**Research shows that diabetes self-management education is effective for improving clinical outcomes and quality of life of people with diabetes but many organizations are not equipped to manage its implementation. This gap can be bridged by peer programs, supported by research from other chronic conditions.
Rotheram-Borus et al. 2012 Pre/post [46]	South Africa	**Peers—**volunteers with diabetes who had lost weight and increased exercise after T2DM diagnosis.	**Actions—**actors (a) led psychoeducational group sessions, (b) facilitated buddy pairs between women with diabetes in order for these women to support each other’s behaviour change via telephone text-communication.	**Targets—**Actions aimed at enhancing self-management for women with diabetes (1 T1D, rest T2DM) who had suffered diabetes symptoms for more than 5 years. Further, actions aimed to facilitate successful buddy pairs, where women with diabetes would support each other’s behaviour change and hereby clinical outcomes.	N/A	**Dose—**Psychoeducational group sessions/informational support meetings—12 weekly meetings. Text-messages—daily. Time period not specified.	**Justification—**Research suggests that peer support can bring significant improvements in chronic disease diagnosis and care. Formative research informed the adaption of the ‘Power to Prevent Program’ to the study setting.
Shen 2008 Pre/post [47]	China	**Peers—**older people with T2DM, living in the same community, non-health professionals. They were very similar to general participants.	**Actions—**actors led a social support and self-efficacy enhancing group activities (SSS-activities). Actors facilitated frequent informal contact and collective peer group meetings.	**Targets—**Older people with T2D (≥ 60 years). Actions are targeted at changing self-management behaviours and subsequent improvement of health outcomes by influencing self-efficacy and social support.	**Temporality—**Informal peer-led SSS-activities started at the same time as basic diabetes information (BDI) by health professionals. Formal SSS-activities 1 week after ended BDI sessions.	**Dose—**SSS activities lasted 12 weeks. Informal contact— at least once a week. Collective group meetings held fortnightly from 5–12th week of study.	**Justification—**Social cognitive theory used as a framework. Research shows that peer education can be used in health promotion and disease prevention programs to lower costs of health education programs. Formative research provided the basis for development of a peer-led T2D self-management program.
Zhong et al. 2015 RCT [51]	China	**Peers**—volunteers, who were retired adults diagnosed with diabetes for a mean of 9.3 years had received training for 3 days in basic skills and DSM. Generally adhered to medication and behavioural management regimens.	**Actions—**actors led educational meetings on DSM, discussion meetings, and organised informal health promotion and support activities such as physical activities.	**Targets—**actions aimed at assisting and encouraging daily diabetes management, providing ongoing social and emotional support, linking community resources and primary care for adults (> 15 years) with T2DM without major co-morbidities.	N/A	**Dose—**educational meetings—12 bi-weekly over 6 months. 1.5-2 h Discussion groups—12 bi-weekly over 6 months Informal activities—not specified.	**Justification—**research suggests that peer support can improve diabetes management. Furthermore, a formative evaluation conducted prior to the study indicated substantial support for the peer-led support program.
Debussche et al. 2018 [50]	Mali	**Peers—**10 PEs from the list of association members: having diabetes, living in the locality, undergoing regular checks with a referent physician, volunteering to deliver educational sessions.	**Actions—**actors led culturally tailored structured patient education (3 courses of 4 sessions) including cardiovascular risk management, food intake, exercise, and blood glucose and insulin management.	**Targets—**actions aimed at evaluating the effectiveness of peer-led self-management education in improving glycaemic control in patients with type 2 diabetes.	N/A	**Dose—**3 courses composed of 4 different thematic sessions (4 ± 10 participants) offered over a period of 3 months (months 1 ± 3, 7 ± 9, and 10 ± 12). The duration of sessions 1.5 ± 2 h	In (LMICs), SME led by community health workers and peers has been reported to make major contributions in the areas of health promotion. However, in the case of (NCDs) such as diabetes, the few studies performed in LMICs have revealed poor outcomes**.**
Less et al. 2010 Pre/post [48]	Jamaica	**CHW—**community health workers classified as local people who were not expected to move away from their communities. Received training and had to complete a standardized questionnaire/test.	**Actions—**actors provided education in DSM through group and one-to-one sessions either at the clinic or in the patients’ homes, when patients could not come to clinic.	**Targets—**actions aimed at increasing knowledge and improve control amongst T2DM patients.	N/A	**Dose—**group sessions and one-to-one interactions—frequency not specified, lasted 6 months.	**Justification—**Due to high net migration rates, training and retaining diabetes educators as part of primary health care system is not feasible. Peer or lay educators may bridge this gap.
Mash et al. 2014 RCT [53]	South Africa	**CHW** (health promoters)**—**lay people employed by community health centres. They were trained (6 day workshop).	**Actions—**actor led sessions of group diabetes education using a guiding style of communication and provided counselling.	**Targets—**actions aimed at enhancing self-management and thus health outcomes for adults with T2DM.	N/A	60 min monthly sessions over 4 months.	**Justification—**poor and limited health infrastructure in LMIC requires task-shifting to cope with the burden of diabetes.
Micikas et al. 2015 Pre/post [49]	Guatemala	**CHW—**nature of these not specified. The actors were selected from a group of community health workers based on interviews. The selected CHW received further training.	**Actions—**actors led education (diabetes club meetings including self-management education, emotional support, physical activities); advocacy (home visits including emotional and medication support); and pre-consults in the clinics with nurse.	**Targets—**actions aimed at improving education, support, and ultimately the health and quality of life of T2DM patients.	N/A	Club meetings—weekly Home visits—weekly Pre-consults—monthly Intervention period is not specified but intervention was evaluated after 4 months.	**Justification—**research shows that community health interventions are an essential component of chronic disease management. Assessments in the intervention villages further underpinned the residents’ strong desire for services provided by community health workers.

#### Actor—who delivers the strategy?

Across the studies, the actors providing the support varied; in eight studies, actors were peers [43–47, 50–52], while in three studies, the support was provided by CHWs [48, 49, 53]. In the peer-led interventions, peers were often volunteers selected based on their knowledge, experience, and adherence to medication and lifestyle changes and thus functioned as role models for patients. In two studies [47, 50], peers were deliberately selected based on their everyday life challenges being similar to those of the patients, while another study did not describe the skill-level of the peers [44]. No description of the CHWs in the three included studies was given, other than they were non-professional health workers (i.e. without a formal health tertiary education) from the local community. In all the studies, the peers/CHWs received training prior to commencing the intervention; most training courses lasted a few days. In one study, Eggermont [45], training lasted 6 weeks. However, in this study, peers also played crucial roles in screening and monitoring clinical measures in patients in addition to providing DSME and DSMS.

#### Actions—which actions do the actors enact?

Across the studies, the peers/CHW aimed at equipping the patients with knowledge, support, and skills to manage their diabetes. Thus, the peers/CHW provided DSMS and DSME. In most of the studies, peers/CHW led the group and discussion meetings [43, 45, 46, 48–52] as well as more informal activities such as organising physical activities and cooking classes. In all studies, peers/CHW provided emotional and/or social support through informal contact with the patient through in-person interactions and/or telephone contact. In two studies, emotional and social support formed the basis of the intervention [44, 47]. Two studies [44, 46] emphasised a deep-grounded one-to-one contact; where peer educators in Baumann et al. [44] were paired with patient peers, peers facilitated the establishment of buddy pairs between patients in Rotheram-Borus et al. [46]. In the remaining interventions, the actions by peers/CHWs targeted both groups and individuals depending on the form of activity. In terms of the types of studies, the interventions were group-based in the four RCTs [50–53] and in five of the seven non-RCT studies; the remaining two non-RCTs were one-to-one interventions [44, 48].

#### Targets of action—who/what are the actors attempting to impact?

All interventions aimed at enhancing emotional and social support for and improving self-care behaviours and management in adults with diabetes. Furthermore, by improving emotional/social support and self-management, the studies aimed to improve clinical outcomes such as glycaemic control, blood pressure, and BMI. Most of the studies targeted T2DM patients exclusively, while two studies did not distinguish between T1DM and T2DM [45, 46]. In two studies, the intervention specifically targeted patients without major comorbidities [51, 52], while the intervention in one study [45] targeted diabetes patients, of whom some also had hypertension. In one study, the intervention targeted older adults (60 years and above) exclusively [47].

#### Temporality—when does the strategy take place?

In all studies, the actions by the actors (i.e. peers/CHW) were commenced following their own training. Only a single study, Shen outlined the relative time from completion of peer/CHW training and the commencement of the interventions [47].

#### Dose—what is the frequency and intensity of the intervention?

The intervention strategies varied in duration and frequency; the strategies ranged from weekly 40-min group discussions [52] to weekly teaching sessions (90–120 min each) and group discussions [47]. The follow-up periods varied between 3 months to 24 months (3 months [47], 4 months [44, 49], 6 months [43, 48, 55], 12 months [50–53], and 24 months [46]).

#### Justification—which (theoretical, empirical, pragmatic) justification is provided for the choice of implementation strategy?

Most of the studies justified the utilisation of a peer/CHW-based intervention for diabetes by referring to existing literature, which highlights peer/CHW-based interventions can contribute to improving chronic conditions. Furthermore, most studies justified the peer/CHW-based intervention by referring to studies showing that such interventions provide a low-cost, flexible means to improve care for chronic conditions in resource-constrained health systems. A few studies also conducted formative research including focus groups and individual interviews in the communities to aid the development of interventions. The formative research illustrated that peer/CHW interventions were desired and/or suited for the given communities [46, 47, 49, 51].

### Implementation outcomes

A comprehensive assessment of implementation outcomes, in terms of acceptability, adoption, appropriateness, feasibility, implementation cost, penetration, and sustainability, is shown in Table 5. Most of the included studies assessed only a few of these outcomes. As an example, implementation cost was only measured in one study [48], while implementation adoption, appropriateness, and penetration were measured in two studies [50, 51]. Acceptability and feasibility were most commonly measured (both were measured in five studies [45, 46, 49–51]).

**Table 5:** Assessment of implementation outcomes of diabetes self-management peer support strategies in LMIC by mode of intervention delivery

Mode of delivery	Domain	Study design	Acceptability	Adoption	Appropriateness	Feasibility	Implementation Cost	Penetration	Sustainability	Available measurement/s
Peer	**Debussche et al. 2018** [50]	**RCT**	**(+)**	**(+)**	**(+)**	**(+)**	**(–)**	**(–)**	**(–)**	**Direct observation**
	**Zhong et al. 2015** [51]	**RCT**	**(+)**	**(+)**	**(+)**	**(+)**	**(–)**	**(+)**	**(+)**	**Focus groups/interviews/report/records**
	**Gagliardino 2014** [52]	**RCT**	**(–)**	**(–)**	**(–)**	**(+)**	**(–)**	**(–)**	**(–)**	**Structured questionnaire/interviews**
	**Assah et al. 2015** [43]	**Non-RCT** **With a control arm**	**(–)**	**(–)**	**(–)**	**(–)**	**(–)**	**(–)**	**(–)**	**Structured questionnaires**
	**Baumann et al. 2014** [44]	**Pre/post**	**(+)**	**(–)**	**(–)**	**(+)**	**(–)**	**(+)**	**(+)**	**Report/phone records/questionnaires**
	**Shen 2008** [47]	**Pre/post**	**(+)**	**(–)**	**(–)**	**(+)**	**(–)**	**(–)**	**(–)**	**Focus groups/report/records/questionnaire**
	**Eggermont 2011** [45]	**Pre/post**	**(–)**	**(–)**	**(–)**	**(–)**	**(–)**	**(–)**	**(–)**	**Structured questionnaire/in-depth interviews**
	**Rotheram-Borus et al. 2012** [46]	**Pre/post**	**(+)**	**(–)**	**(–)**	**(+)**	**(–)**	**(–)**	**(–)**	**Structured questionnaire/interviews**
NCHWS	**Mash et al. 2014** [53]	**RCT**	**(–)**	**(–)**	**(–)**	**(–)**	**(–)**	**(–)**	**(–)**	**Interviews/report/records/questionnaire**
	**Micikas et al. 2014** [49]	**Pre/post**	**(+)**	**(–)**	**(–)**	**(–)**	**(–)**	**(–)**	**(–)**	**Focus groups/structured questionnaires**
	**Less et al. 2010** [48]	**Pre/post**	**(–)**	**(–)**	**(–)**	**(–)**	**(+)**	**(–)**	**(+)**	**Structured questionnaire**

(+) measured, (–) none, *NPCHW* non-professional community health workers

### Diabetes-related outcomes

The diabetes-related outcomes described by study design, (RCT and non-RCT design) are summarised in Table 6 and detailed below.

**Table 6:** Summary of intervention effects on clinical, behavioural and psychological outcomes by study design

Authors, year	Debussche et al., 2018 [50]	Zhong et al. 2015 [51]	Gagliardino 2014 et al. [52]	Mash et al. 2014 [53]	Assah et al. 2015 [43]	Micikas et al. 2014 [49]	Baumann et al. 2014 [44]	Shen 2008 [47]	Eggermont 2011 [45]	Rotheram-Borus et al. 2012 [46]	Less et al. 2010 [48]
Design	RCT	RCT	RCT	RCT	Non-RCT with control	Pretest-posttest (one group)	Pretest-posttest (one group)	Pretest-posttest with comparison group	Pretest posttest (one group)	Pretest-posttest (one group)	Pretest-posttest with comparison group
Sample size	*n* = 151 *C* 76 *I* 75	*n* = 229 *C* 94 *I* 135	*n* = 198 *C* 105 *I* 93	*n* = 1570 *C* 860 *I* 710	*n* = 200 *C* 96 *I* 96	*n* = 100	*n* = 46	*n* = 181 *C* 89 *I* 92	*n* = 3078	*n* = 19	*n* = 318 *C* 159 *I* 159
Duration of diabetes (year)	NR	9.3	6	NR	NR	NR	6.7	NR	NR	NR	5–21
Follow-up (months)	12	12	12	12	6	4	4	3	24	6	6
HbA1C	↓	NR	NR	↔	↓	↓	↓	NR	NR	NR	↓
FBG/PPG	NR	↓	NR	NR	NR	NR	NR	NR	↓	NR	NR
BP	NR	↓	NR	↓	NR	NR	↓ DBP	NR	↓	NR	NR
BMI	↓	↓	↔	↔	NR	↔	NR	NR	↔	↔	↔
Diabetes symptoms	NR	NR	↔	NR	NR	NR	NR	NR	NR	NR	NR
Clinical visits/hospitalis-ation	NR	NR	NR	NR	NR	NR	NR	↔	NR	NR	NR
Self-management activities	NR	↔	NR	↔	↑	NR	↔	↑	NR	NR	NR
Physical activity	NR	NR	↔	NR	NR	↔	NR	NR	NR	NR	NR
Self-efficacy	NR	↑	NR	↔	NR	NR	NR	↑	NR	NR	NR
Diabetes knowledge	NR	↑	↑	NR	NR	↑	NR	NR	NR	NR	NR
Depression	NR	NR	↔	↔	NR	NR	NR	↔	NR	↔	NR
Social support	NR	NR	NR	NR	NR	NR	NR	↑	NR	↑	NR
Quality of life	NR	NR	NR	↔	NR	NR	NR	↔	NR	NR	NR

*↔* no statistical significant differences, ↑ significant increase, ↓ significant decrease, *NR* not reported, *HbA1C* glycated hemoglobin, *FBG/PPG* fasting glucose and 2-h postprandial glucose, *BP* blood pressure, *BMI* body mass index, *NR* not reported

### Randomised controlled trials

#### Clinical outcomes

##### HbA1c

Of the 4 RCTs, only 2 studies (Mash et al. and Debussche et al.) assessed HbA1c. In one study, the mean reduction in HbA1c of 1.05% between intervention and control groups was both statistically and clinically significant [50].

##### Fasting glucose and 2-h postprandial glucose (FPG/PPG)

Zhong et al. was the only RCT which examined changes in fasting glucose levels. The study showed a reduction from 7.68 to 6.76 mmol/L for the intervention group, while those in the control groups exhibited a slight increase from 6.38 to 6.66 mmol/L. [51]. The difference between these two patterns was statistically significant (*p* < .001), but the authors did not report on its clinical relevance.

##### Blood pressure

Two of three RCTs, Zhong et al. and Mash et al. [51, 53], reported on blood pressure. These studies found significant reductions in systolic blood pressure (SBP) and diastolic blood pressure (DBP) in the peer/CHW-led interventions compared to the control groups.

Zhong et al. [51] reported a significant reduction in SBP in their interventional group (136–128 mmHg) compared to the control group (130–131 mmHg) and in DBP (intervention 82.5–79.1 mmHg; control 79.0–78.6 mmHg). Mash et al. [53] reported a weighted mean SBP reduction of 4.65 mmHg (95% CI − 9.18 to − 0.12), which was not statistically significant. Further, a weighted mean DBP reduction of − 3.30 mmHg (95% CI − 5.35 to − 1.26) was statistically significant.

##### Body mass index

Two RCTs measured BMI, which was reduced in the peer-led intervention groups [50, 51].

##### Diabetes symptoms

Only Gagliardino et al. reported changes in classical diabetes symptoms (polydipsia, polyuria, polyphagia, pruritus, and asthenia). The symptoms were statistically significantly reduced in both control and intervention groups between baseline and 12 months with no significant differences between control and intervention reductions [52]. The authors did not report whether these findings were clinically relevant.

#### Behavioural health outcomes

##### Self-management care outcome

Only two RCT studies reported on self-management care activities. Zhong et al. and Mash et al. [51, 53] found no significant differences in self-management practices relating to diet, physical activity, glucose monitoring, and medication adherence.

##### Physical activity

Two of the three RCTs reported on physical activity. Gagliardino et al. [52] demonstrated a significant reduction in the number of participants practicing regular physical activity in control (56 to 37%, *p* = 0.0006), but not the COMP-DSMP intervention group (69 to 60%, *p* = 0.221). Notably, there was a significant difference in the reduction (in %) between the two groups (19% versus 9%, *p* = 0.035). Zhong et al. [51] reported no improvement in self-reported physical activity.

#### Psychological health outcomes

##### Self-efficacy

Although self-efficacy for diabetes management was measured using different scales, two RCT studies reported increased self-efficacy in the intervention group compared to the control group when measured at 6 to 12 months. Zhong et al. [51] found significant improvements in self-efficacy with the intervention in two out of six sites, where a COMP-DSMP was implemented. However, Mash et al. [53] reported the COMP-DSMP intervention did not improve psychological health outcomes including self-efficacy.

##### Diabetes knowledge

Two RCT studies reported on diabetes knowledge. Zhong et al. [51] found statistically significant improvements at 12 months’ follow-up in a knowledge domain made up of twelve items (four concerning glucose, three concerning diabetic complications, two concerning diet, and three concerning insulin). However, the control group’s knowledge diminished from baseline. Gagliardino et al. showed that the attendees’ knowledge increased significantly (*p* < 0.01) in both groups, but without any significant difference between intervention and control groups.

##### Depression, diabetes-related distress, and quality of life

Gagliardino et al. reported on diabetes-related distress, while Mash et al. reported on depression. Neither study reported significant differences in these measurements in the intervention and control groups at baseline to 12 months [52, 53]. Zhong et al. did not report on any of these measures, and none of the RCTs reported on quality of life.

### Pre-test/post-test and non-RCTs

#### Clinical outcomes

##### HBA1c

Four studies, Assah et al., Micikas et al., Baumann et al., and Less et al. [43, 44, 48, 49] reported HbA1c as an outcome measure. Assah et al. [43] reported a greater reduction in HbA1c in the peer-led intervention than the control group (difference = − 1.7%, 95% CI − 2.2 to − 1.3%, *p* < 0.001). The other three studies with significant improvements were quasi-experimental in design. Micikas et al. and Baumann et al. [44, 49] reported the mean HbA1c decreased from 10.1% and 11.1% at baseline to 8.9% and 8.3% at 4 months (*p* = 0.01 and *p* = 0.005) respectively. Less et al. reported a reduction of 0.6% in mean HbA1c in the intervention group between baseline and 6-month values and an increase of 0.6% in the control group, with the difference being statistically significant after controlling for potential confounders (*p* < 0.05) [48].

##### Fasting blood glucose levels

Only Eggermont et al. reported on blood glucose outcomes. The study demonstrated a statistically significant reduction in fasting blood glucose (10.0 to 7.7 mmol/l, *p* < 0.001) 2 years after baseline [45].

##### Blood pressure

Two studies reported on blood pressure. Eggermont et al. identified significant reductions in systolic (134 to 124 mmHg, *p* < 0.001) and diastolic (85 to 77 mmHg, *p* < 0.001) blood pressure [45]. In contrast, Baumann et al. [44] reported that diastolic (85–76 mmHg, *p* < 0.001) but not systolic (146–140 mmHg, *p* = 0.25) blood pressure decreased significantly in the intervention group compared to the control group.

##### BMI

There was no difference found between- or within-group changes in BMI in the four studies where BMI was assessed [45, 46, 48, 49].

#### Behavioural health outcomes

##### Self-management care outcome

Three studies reported on self-management care activities. Assah et al. [43] found a significantly increased level of self-care activities in the intervention compared to the control group (*p* < 0.001). Baumann et al. [44] reported that the adherence to the eating plan improved from pre- to post-intervention (*p* < 0.005), which was measured regarding a ‘healthy eating index’ created by the authors. However, there were no significant pre/post-intervention changes in physical activity, missed medication, helpfulness of social support, emotional well-being, confidence, and barriers to self-care. Shen [47] reported that overall self-management activities were significantly higher in the intervention group compared to the control group. However, self-management of diet and medication did not differ significantly between the two groups at 4 and 12 weeks. These analyses were adjusted for baseline variables and multiple comparisons.

Only Shen [47] reported a reduction in the number of visits to a doctor (MD − 0.73, *p* = 0.03) and a community health centre (MD − 0.60, *p* = 0.03) in the intervention compared to the control group. However, there were no significant differences in the number of visits to the emergency room, the frequency of hospitalisation, or the days of hospitalisation between the groups at 4 or 12 weeks.

##### Physical activity

One study, Micikas et al. [49], reported on physical activity. The study did not observe significant changes in the proportion of patients who exercised for ≥ 30 min per day after 4 months of the intervention (20 to 18%, *p* = 0.811).

#### Psychological health outcomes

##### Self-efficacy

One study reported on self-efficacy. Baumann et al. [44] measured various aspects of self-efficacy including overall self-efficacy and self-efficacy relating to diet, exercise, medication use, blood glucose testing, foot care, and hyperglycaemia/hypoglycaemia. At 4 weeks and 12 weeks, overall self-efficacy (both *p* < 0.021) and self-efficacy relating to blood glucose testing (both *p* < 0.005), foot-care (both *p* < 0.001), and hyper/hypoglycaemia (both *p* < 0.001) were significantly higher in the intervention compared to the control group.

##### Diabetes knowledge

One study reported on diabetes knowledge. Micikas et al. [49] reported that the intervention (over 4 months) significantly improved diabetes knowledge about targets for HbA1C (6 to 42%, *p* = 0.001), fasting blood glucose (19 to 87%, *p* = 0.001), and the foods that raise blood glucose levels (13 to 31%, *p* = 0.032). However, knowledge about the impact of emotions on blood glucose levels did not improve (*p* = 0.687).

##### Depression, diabetes distress, and quality of life

Two studies reported on these measures. Shen [47] measured depressive status subdivided into ‘overall depressive status’, ‘unhappy status’, ‘somatic status’, ‘interpersonal status’, and ‘depressed affect status’. The mean score for ‘unhappy status’ decreased significantly in the intervention group between 4 and 12 weeks (*p* = 0.037), while it did not change in the control group (*p* = 0.26). However, there were no significant differences between the intervention and control groups regarding ‘overall depressive status’, ‘somatic’, ‘interpersonal’, and ‘depressed affect status’. Rotheram-Borus et al. [46] evaluated diabetes distress in terms of coping skills, which was significantly improved between baseline and 3 months, but not between 3 and 6 months. Furthermore, spiritual hope decreased between 3 and 6 months (*p* < 0.01).

##### Social support

Two studies reported on social support. Shen [47] identified that the intervention group had significantly higher overall social support (both *p* < 0.001), information and emotional support (both *p* < 0.001), positive interaction (both *p* < 0.001), and affectionate support (both *p* < 0.001) compared to the control group assessed at 4 and 12 weeks. However, the peer intervention failed to improve tangible support significantly (include assisting with transportation, helping with household chores, helping to prepare food, providing physical care, and providing financial help) at the same time intervals 4 and 12 weeks. Rotheram-Borus et al. reported improvements in social support (*p* < 0.01) and positive-action coping style (*p* < 0.01) after 3 months [46].

##### Implementation fidelity of all the included studies

Implementation fidelity refers to the extent to which a proposed intervention is enacted as designed. This measure is essential to determine to which extent the intervention in question is the primary mechanism underlying any changes observed [42, 54]. Table 7 summarises the adherence, moderators, and assessment fidelity of the included studies in this review. The moderators of fidelity refer to factors which may influence or moderate the degree of fidelity with which an intervention is implemented such as intervention complexity, facilitation strategies, quality of delivery, and participant responsiveness [54, 55]. We were able to monitor fidelity in four studies, which had published protocols [47, 50, 51, 53]. The most frequently used indicator of fidelity was adherence (to the content, frequency, and duration of the intervention) [47, 51, 53]. Four studies [47, 50, 51, 53] refer to facilitating strategies to increase the implementation quality. In one study [53], the quality of the program delivery was used as a moderator. Overall, three studies [47, 51, 53] used a questionnaire or interviews completed by the participants and providers. Three studies [47, 50, 53] combined a direct observation, recording of sessions, and self-reported measures (questionnaire or interviews completed by the participants and providers) to assess the adherence to the program content.

**Table 7:** Assessment of elements of implementation fidelity

Study by mode of intervention delivery	Adherence				Moderators				Assessment					
	Content	Coverage	Frequency	Duration	Intervention complexity	Facilitation strategies	Quality of delivery	Participant responsiveness	Direct observation	Audio/video tap	Provider questionnaire or checklist	Provider interview	Participants questionnaire	Participants interview
Peers														
Debussche et al. 2018 [50]	**✓**	**NA**	**✓**	**✓**	**NA**	**✓**	**NA**	**✓**	**✓**	**NA**	**NA**	**NA**	**NA**	**NA**
Zhong et al. 2015 [51]	**✓**	**NA**	**✓**	**✓**	**✓**	**✓**	**NA**	**✓**	**NA**	**✓**	**NA**	**✓**	**NA**	**✓**
Gagliardino et al. 2014 [52]	NA	NA	NA	NA	NA	NA	NA	NA	NA	NA	NA	NA	NA	NA
Baumann et al. 2014 [44]	NA	NA	NA	NA	NA	NA	NA	NA	NA	NA	NA	NA	NA	NA
Shen 2008 [47]	✓	NA	✓	✓	NA	✓	NA	✓	✓	✓	✓	✓	✓	✓
Eggermont 2011 [45]	NA	NA	NA	NA	NA	NA	NA	NA	NA	NA	NA	NA	NA	NA
Rotheram-Borus et al. 2012 [46]	NA	NA	NA	NA	NA	NA	NA	NA	NA	NA	NA	NA	NA	NA
Assah et al. 2015 [43]	NA	NA	NA	NA	NA	NA	NA	NA	NA	NA	NA	NA	NA	NA
NCHWs														
Mash et al. 2014 (47)	**✓**	**NA**	**✓**	**✓**	**✓**	**✓**	**✓**	**✓**	**✓**	**✓**	**-**	**✓**	**✓**	**✓**
Micikas et al. 2014 [49]	NA	NA	NA	NA	NA	NA	NA	NA	NA	NA	NA	NA	NA	NA
Less et al. 2010 [48]	NA	NA	NA	NA	NA	NA	NA	NA	NA	NA	NA	NA	NA	NA

*IF* implementation fidelity, *NA* not assessed in the primary papers

## Discussion

This systematic review found that COMP-DSMPs were inconsistently associated with improvements in clinical [43, 47, 48, 50–53] behavioural [43, 44, 52], and psychological [43, 44, 51–53] outcomes in LMIC. There was high variability in reported outcomes. As a consequence, meaningful meta-analysis or comparisons were not possible. The included studies only assessed short-term outcomes, and no ‘hard-endpoints’ in terms of co-morbidity, microvascular, macrovascular events, and mortality were reported.

Most included studies were of low-quality design with significant risks of bias particularly in relation to the blinding of outcomes. Furthermore, the majority of the non-RCT studies did not address issues surrounding selection bias or gave insufficient information regarding the selection process. The strategies employed were often not described in much detail and were poorly or not assessed regarding implementation fidelity. Finally, most of the studies reporting on clinical outcomes only evaluated the outcomes in terms of statistical significance and not clinical relevance. These results are discussed here in terms of implications for health care delivery, as well as implications for future design and implementation of studies of COMP-DSMPs in diabetes care delivery in LMIC.

Current literature, mainly from HIC, supports the findings from this review that effects of COMP-DSMPs are equivocal. A systematic review by Webel et al. reported major heterogeneity between studies both in outcomes and designs [55], while Dale et al. reported that peer-support seemed to benefit some adults living with diabetes, but suggested that the evidence was inconsistent and inadequate to support firm recommendations [56]. Large RCTs in HIC [57, 58] have also reported modest benefits for some, but not all outcomes assessed.

Throughout our analysis of the nature of the implementation strategies, we relied on the framework developed by Proctor et al. [41]. While most of the studies touched on five of the six dimensions included in the taxonomy adapted for this study, none directly followed the Proctor framework and thus did not readily lend themselves to being evaluated using this framework. Further, none of the studies described temporality. This is at odds with Proctor’s suggestion that temporality be considered by researchers planning implementation/intervention programs and applied systematically across study populations, unless otherwise stated, to ensure that the experimental conditions are the same. Temporality can be a critical factor contributing to the effectiveness of an implementation strategy, because peers/CHWs’ skills, knowledge, and engagement may decrease over time if not employed.

Few existing studies have employed Proctor’s framework. Yet studies such as Powel et al. [59] illustrate the value of using such a standardised framework when they adopted the framework to report on a diabetes quality improvement intervention in its original commercial care setting and in community health care centres, in which actors, action, temporality, and dose were adapted to fit the local context [59]. The models of peer/CHW-led program need to be further explored, especially given the inevitability of a professional healthcare workforce shortage in LMIC.

Furthermore, COMP-DSMPs have the potential to fulfil the ideals of ‘triple aims of health care’ defined by the Institute for Healthcare Improvement, which involves improving a patient’s experiences of care, improving the health of populations, and reducing per capita costs of care. Thus, COMP-DSMPs for diabetes deserve more attention [60]. Future studies should include an assessment of cost-effectiveness due to the limited data on this aspect of COMP-DSMPs. A single study in HIC from the UK revealed no significant differences in the final cost-effectiveness endpoints for a group-based peer support intervention for type 2 diabetes in general practice [61]. Mash et al. found that a structured group education program delivered by health promotors at primary care clinics in South Africa for the management of type 2 diabetes was cost-effective [63]. Ideally, it would be important to assess cost-effectiveness in terms of traditional hard outcomes such as mortality and micro- and macro-vascular complications, but the longer length of follow-up and large sample sizes are likely to be costly [62].

This systematic review has limitations. Of the 11 studies included, only 4 were RCTs and 7 were non-RCTs, including uncontrolled before and after studies. Because of the scarcity of RCTs from LMICs, these were included here to provide an overview of such studies previously done and to emphasise the limitations of such studies.

In conclusion, there is only limited and low level of evidence for benefit from peer support (in its broadest sense, here including CHWs) in diabetes care outcomes. We recommend that future studies consider using a framework such as the MRC framework for the development and evaluation of complex interventions to inform the evidence base on the contribution of COMP-DSMP in LMIC.

## Supplementary information

**Additional file 1.** Pubmed search strategy.

**Additional file 2.** Cochrane risk of bias tool.

## Abbreviations

LMIC: Low- and middle-income countries
CHWs: Community health workers
COMP-DSMP: Community-based peer-led diabetes self-management programs
