# Supplementary material for: The effectiveness of peer and community health worker-led self-management support programs for improving diabetes health-related outcomes in adults in low- and-middle-income countries: a systematic review
Source: Syst Rev. 2020 Jun 6;9:133. doi: 10.1186/s13643-020-01377-8 (PMC7275531; doi:10.1186/s13643-020-01377-8)
Supplement: Supplementary file 1 — Additional file 1. Pubmed search strategy. [file 13643_2020_1377_MOESM1_ESM.docx]

Addiitonal File_1. Pubmed Search Strategy

((("Diabetes Mellitus"[Mesh] OR ("diabetes mellitus"[MeSH Terms] OR ("diabetes"[All Fields] AND "mellitus"[All Fields]) OR "diabetes mellitus"[All Fields])) AND ((((((((((((((((peer[All Fields] AND based[All Fields] AND interventions[All Fields]) OR (peer-led[All Fields] AND interventions[All Fields])) OR (peer[All Fields] AND ("education"[Subheading] OR "education"[All Fields] OR "educational status"[MeSH Terms] OR ("educational"[All Fields] AND "status"[All Fields]) OR "educational status"[All Fields] OR "education"[All Fields] OR "education"[MeSH Terms]))) OR "Social Support"[Mesh]) OR peers[All Fields]) OR (peer[All Fields] AND support[All Fields])) OR (peer[All Fields] AND ("counselling"[All Fields] OR "counseling"[MeSH Terms] OR "counseling"[All Fields]))) OR "Self-Help Groups"[Mesh]) OR (("population groups"[MeSH Terms] OR ("population"[All Fields] AND "groups"[All Fields]) OR "population groups"[All Fields] OR "group"[All Fields]) AND support[All Fields])) OR (("population groups"[MeSH Terms] OR ("population"[All Fields] AND "groups"[All Fields]) OR "population groups"[All Fields] OR "group"[All Fields]) AND ("education"[Subheading] OR "education"[All Fields] OR "educational status"[MeSH Terms] OR ("educational"[All Fields] AND "status"[All Fields]) OR "educational status"[All Fields] OR "education"[All Fields] OR "education"[MeSH Terms]))) OR (peer[All Fields] AND leader[All Fields])) OR (lay[All Fields] AND ("health educators"[MeSH Terms] OR ("health"[All Fields] AND "educators"[All Fields]) OR "health educators"[All Fields]))) OR (lay[All Fields] AND ("occupational groups"[MeSH Terms] OR ("occupational"[All Fields] AND "groups"[All Fields]) OR "occupational groups"[All Fields] OR "workers"[All Fields]))) OR (lay[All Fields] AND ("health"[MeSH Terms] OR "health"[All Fields]) AND advisor[All Fields])) OR "Community Health Workers"[Mesh]) OR ("community health workers"[MeSH Terms] OR ("community"[All Fields] AND "health"[All Fields] AND "workers"[All Fields]) OR "community health workers"[All Fields] OR ("community"[All Fields] AND "health"[All Fields] AND "worker"[All Fields]) OR "community health worker"[All Fields]))) AND (((("poverty"[MeSH Terms] OR "poverty"[All Fields] OR ("low"[All Fields] AND "income"[All Fields]) OR "low income"[All Fields]) AND countries[All Fields]) OR (middle[All Fields] AND ("income"[MeSH Terms] OR "income"[All Fields]) AND countries[All Fields])) OR (low-middle[All Fields] AND ("income"[MeSH Terms] OR "income"[All Fields]) AND countries[All Fields]))) AND (((((interventions[All Fields] OR "Randomized Controlled Trial"[Publication Type]) OR RCT[All Fields]) OR "Controlled Clinical Trial"[Publication Type]) OR CCT[All Fields]) OR experiment[All Fields] AND "adult"[MeSH Terms]) AND ("2000/01/01"[PDAT] : "2019/12/31"[PDAT])
