# Supplementary material for: The effectiveness of peer and community health worker-led self-management support programs for improving diabetes health-related outcomes in adults in low- and-middle-income countries: a systematic review
Source: Syst Rev. 2020 Jun 6;9:133. doi: 10.1186/s13643-020-01377-8 (PMC7275531; doi:10.1186/s13643-020-01377-8)
Supplement: Supplementary file 2 — Additional file 2. Cochrane risk of bias tool. [file 13643_2020_1377_MOESM2_ESM.docx]

**Additional file 2**

**Cochrane Risk of Bias Tool**

| Domain | Description | High Risk of Bias | Low Risk of Bias | Unclear Risk of bias | Reviewers assessment | Reviewers comments |
| --- | --- | --- | --- | --- | --- | --- |
| \| *Selection bias* ***Random sequence generation*** \| \| --- \| | Described the method used to generate the allocation sequence in sufficient detail to allow an assessment of whether it should produce comparable groups | Selection bias (biased allocation to interventions) due to inadequate generation of a randomized sequence | Random sequence generation method should produce comparable groups | Not described in sufficient detail | **High Low Unclear** |  |
| *Selection bias Allocation concealment* | Described the method used to conceal the allocation sequence in sufficient detail to determine whether intervention allocations could have been foreseen before or during enrollment | Selection bias (biased allocation to interventions) due to inadequate concealment of allocations prior to assignment | Intervention allocations likely could not have been foreseen in before or during enrollment | Not described in sufficient detail | **High Low Unclear** |  |
| *Reporting bias Selective reporting* | Stated how the possibility of selective outcome reporting was examined by the authors and what was found   \|  \| \| --- \| | Reporting bias due to selective outcome reporting | Selective outcome reporting bias not detected | Insufficient information to permit judgment† | **High Low Unclear** |  |
| *Performance bias Blinding (participants and personnel)* | Described all measures used, if any, to blind study participants and personnel from knowledge of which intervention a participant received. Provided any information relating to whether the intended blinding was effective. | Performance bias due to knowledge of the allocated interventions by participants and personnel during the study. | Blinding was likely effective. | Not described in sufficient detail | **High Low Unclear** |  |
| *Detection bias Blinding (outcome assessment* | Described all measures used, if any, to blind outcome assessors from knowledge of which intervention a participant received. Provided any information relating to whether the intended blinding was effective | Detection bias due to knowledge of the allocated interventions by outcome assessors. | Blinding was likely effective. | Not described in sufficient detail | **High Low Unclear** |  |
| *Attrition bias Incomplete outcome data* | Described the completeness of outcome data for each main outcome, including attrition and exclusions from the analysis. Stated whether attrition and exclusions were reported, the numbers in each intervention group (compared with total randomized participants), reasons for attrition/exclusions where reported. | Attrition bias due to amount, nature or handling of incomplete outcome data. | Handling of incomplete outcome data was complete and unlikely to have | Insufficient reporting of attrition/exclusions to permit judgment (e.g., number randomized not stated, no reasons for missing data provided) | **High Low Unclear** |  |
